# Predictors for falling within six months after surgery in patients with hemiarthroplasty after an acute femoral neck fracture

**DOI:** 10.1038/s41598-026-35974-9

**Published:** 2026-01-19

**Authors:** Ariena J. Rasker, Lisa Berghorst, Nienke W. Willigenburg, Maria C. J. M. Tol, Rudolf W. Poolman, Hanna C. Willems

**Affiliations:** 1 Department of Orthopedics, Joint Research, OLVG Hospital, Amsterdam, the Netherlands; 2 Department of Orthopedics, Leiden University Medical Center, Leiden, the Netherlands; 3 Department of Internal Medicine and Geriatrics, Amsterdam UMC, Location University of Amsterdam, Amsterdam, the Netherlands

**Keywords:** Hip Fracture, Hemiarthroplasty, Predictors, Falling, Risk factors, Clinical trials

## Abstract

For patients who underwent hip hemiarthroplasty, usually due to a femoral neck fracture from a fall, subsequent falls can have life-threatening consequences. This study identified predictors for falling in patients from a clinical trial comparing the direct lateral and posterolateral approach. Patients were included if they participated in the APOLLO trial. This secondary analysis divided patients into ‘fallers’ during 6 months follow-up, ‘non-fallers,’ and ‘unknown.’ Two logistic regression analyses were conducted: one using variables collected pre-surgery and one using variables available at discharge. The outcome variable 'fallen within 6 months after hemiarthroplasty’ was dichotomous. Among the patients with known fall status, 219 (48%) fell one or more times and 240 (52%) were non-fallers. Fall status was unknown for 384 (46%) of the 843 APOLLO trial participants. Falling was associated with five pre-surgery variables: impaired mobility, ADL difficulties, neurological and pulmonary comorbidities, and dementia. At discharge, falling was associated with impaired mobility, ADL difficulties, and in-hospital complications. The pre-surgery model had an R^2^ of 0.217 and an Area Under the Curve (AUC) of 0.730 (95% CI 0.7–0.8), while the discharge model had an R^2^ of 0.216 and an AUC of 0.709 (95% CI 0.7–0.8). Both models showed moderate predictive ability for falling. This study highlights the substantial incidence of falls after hemiarthroplasty for femoral neck fractures and identified predictors for falling within six months. The multidisciplinary team should prioritize fall prevention, and future research should validate prediction models and assess whether fall prevention strategies can effectively reduce falls.

**Supplementary Information:**

The online version contains supplementary material available at 10.1038/s41598-026-35974-9.

## Introduction

Despite various tools have been developed to reduce fall risk, the rising prevalence of falls remains a substantial public health issue, especially among older adults^1,2^. The increasing incidence of falls not only threatens the physical well-being of individuals but also has a profound impact on their overall quality of life^3–5^. Falling is associated with adverse outcomes, including injuries, fear of falling, and social isolation. Especially for individuals who have experienced a fall before and underwent hemiarthroplasty surgery, a subsequent fall can carry life-threatening consequences^6^.

Research shows that 61.5% of patients with a recent fracture experience a fall within three years^7^. The fall risk is highest in the first year after any fracture^8^, with a likely higher risk for those who have sustained a hip fracture, as hip fractures often lead to reduced mobility. Geriatric patients are particularly vulnerable, often having multiple chronic health conditions, reduced mobility, and increased dependency. Preventing subsequent falls and identifying patients at risk is crucial, as repeated falls within the first year after a hip fracture can adversely affect recovery and reduce social participation^9^.

While some studies have investigated predictors for falling in the hip fracture population^10^, little is known about predictors for falling specifically in patients with hip hemiarthroplasty, and over the longer period after rehabilitation. This group, often older adults with multiple comorbidities, is at heightened risk for falls and mobility limitations after surgery. Although research has explored the risk of second fractures^11–14^, there is limited research on the risk of falling separately. Additionally, while Tol et al. (2024) found no difference in fall frequency between the Direct Lateral Approach (DLA) and Posterolateral Approach (PLA) for hemiarthroplasty, other predictors influencing fall risk remain unclear. This study aimed to identify predictors for falling after hip hemiarthroplasty in patients who participated in a trial comparing the DLA and PLA.

## Methods

### Study design

We performed post-hoc analyses for a randomized trial comparing the DLA and PLA for hip hemiarthroplasty surgery after an acute femoral neck fracture.

### Participants

Patients participated in the APOLLO trial^15^; a multicenter Randomized Controlled Trial (RCT) alongside a Natural Experiment (NE) and economic evaluation (NCT04438226, retrospectively registered on June 11, 2019). The APOLLO trial compared two surgical approaches: DLA and PLA. In the RCT, patients were randomized for DLA or PLA. In the NE, the preference and experience of the orthopaedic surgeon determined whether the PLA or DLA approach was used for hemiarthroplasty. For these secondary analyses, patients were included if they provided written informed consent for the RCT or NE of the APOLLO trial.

### Variables and outcome measures

Falls were ascertained through surveys conducted at 3 and 6 months post-surgery, in which patients were asked whether they had fallen during the preceding period and, if so, how many times. Surveys were distributed by post or email, with most patients preferring post. If a survey question was unanswered, researchers made up at least one attempt to contact the patient by phone to complete the missing data; otherwise, the data point was classified as missing. Additionally for this study, the medical health records of participants were reviewed to identify visits to the emergency room due to a fall at the hospital they underwent surgery. Fall-related injuries were identified through a survey question asking patients whether they had sustained an injury as a result of a fall, and from a review of their medical records.

We divided the study population into three groups. Patients who had reported a fall, or were admitted to the emergency room due to a fall within 6 months after hemiarthroplasty were classified as ‘fallers’. Those who explicitly reported that they had not fallen and were not admitted to the emergency room due to a fall within 6 months were classified as ‘non-fallers’. Additionally, we categorized a third group classified as ‘unknown’ for patients where it was unclear whether they fell based on surveys or medical health records. Patients who deceased within 3 or 6 months and had not yet completed the survey were also placed in the unknown group, because it remains unknown whether they would have fallen if they had not died. If a patient fell before death, they were categorized as ‘fallers.’

The following variables were collected before hemiarthroplasty surgery: age, gender, BMI, number of prescribed medication (dichotomized as polypharmacy for five or more medications), ASA classification, cardiac comorbidity, pulmonary comorbidity, neurological comorbidity, dementia according to clinical judgment of physician, living situation (home and independent, home with daily help, assisted living facility, rehabilitation center, nursing home), mobility score (no walking aids, outside with one cane, outside with two canes or walker, never outside without help, no functionality of lower extremity), and activities of daily living functionality (Katz ADL score)^16^.

The following variables were collected after surgery and before discharge: length of acute hospital stay, surgical approach (DLA or PLA), in-hospital complications, and discharge destination (home and independent, home with daily help, assisted living facility, rehabilitation center, nursing home, hospice). In-hospital complications were identified through medical record reviews and included conditions such as anemia, pneumonia, infection, and delirium. Living situation, mobility score, and discharge destination were analyzed as ordinal variables, assuming that each level contributes equally to the risk of falling. The outcome variable 'fallen within 6 months after hemiarthroplasty’ was dichotomous.

### Sample size and power considerations

Following the rule of thumb of 10 events per degree of freedom and 219 fallers in our comprehensive database, the analysis had sufficient power to explore all potential predictor variables^17^.

### Statistics

Using descriptive statistics, baseline characteristics were summarized for fallers, non-fallers, and those with unknown fall status. Missing data were generally low (≤ 7.1%) for most variables, though BMI and Katz ADL score had higher missing rates (up to 19.6%). A comparison of complete and incomplete cases showed no meaningful differences in baseline characteristics, suggesting that missing data were likely missing completely at random (MCAR). Therefore, multiple imputation was not performed. Given the low overall proportion of missing data, its impact on outcomes is expected to be minimal.

Univariable logistic regression was used to determine the association between predictors and fall risk, with results expressed as odds ratios (OR) and significance defined as p < 0.05. To assess multicollinearity, Spearman’s correlation coefficients were calculated, as well as the variance inflation factors (VIF). All candidate predictors were then entered into a multivariable logistic regression analysis. To address potential confounding, we included demographic variables (age, gender), proxies for health status and medication burden (ASA classification and polypharmacy), baseline functional status (mobility score and Katz ADL), living situation, and comorbidity domains (cardiovascular, neurological, pulmonary, and dementia) as candidate predictors in the multivariable models. In the discharge model, discharge destination and in-hospital complications were additionally considered.

A backward selection procedure with Akaike’s Information Criterion (AIC) was applied to identify the most relevant predictors^18,19^. Unlike strict reliance on Wald p-values, which only assess the precision of individual predictors, the AIC is equivalent to the selection of p < 0.157 for predictors with 1 degree of freedom. For variables with multiple degrees of freedom, the AIC evaluates their combined contribution to the model. This approach ensured that no potentially relevant predictors were prematurely excluded^20^ while balancing model complexity with explanatory power.

We conducted two logistic regression analyses using two different sets of variables to address different clinical scenarios. The first analysis included only pre-operative variables, relevant for making decisions before surgery, such as choosing the surgical approach. The second analysis incorporated all variables available up to discharge, providing a more comprehensive view for predicting falls from the time of discharge. For both analyses, only patients with known fall status (fallers and non-fallers) were included. Patients classified as ‘unknown’ were excluded, as fall occurrence could not be reliably ascertained. Consequently, the reported associations apply to the observed subset with known fall status. Model performance was evaluated using Nagelkerke R^2^ and the Area Under the Receiver Operating Curve (AUC). All analyses were performed using SPSS (Version 29.0; IBM Corp, Armonk, NY, USA).

## Results

### Study population

**Fig. 1:**
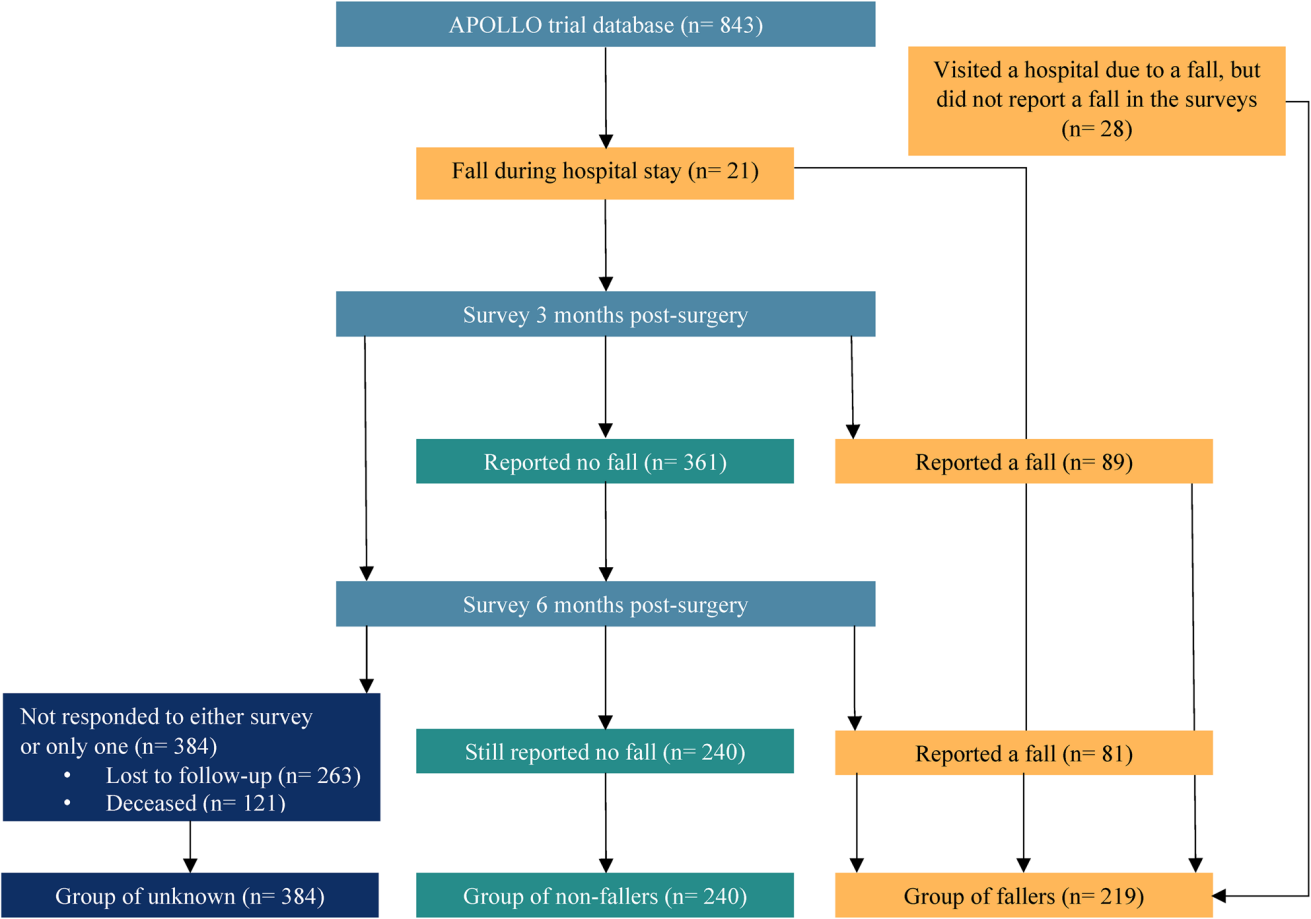
Flowchart of the study population. This flowchart illustrates the classification of the APOLLO trial study population into three groups: fallers, non-fallers, and unknown fall status. The groups are defined using data from questionnaires and patient records. Patients whose fall status is unknown are represented by dark blue blocks, non-fallers are represented by green blocks, and fallers are represented by yellow blocks. The light blue blocks represent the study time points.

Among the patients with known fall status, 219 (48%) fell one or more times and 240 (52%) were non-fallers. Fall status was unknown for 384 (46%) of the 843 APOLLO trial participants (Fig. 1). Of the total study population, 121 patients (14.4%) were deceased within 6 months after surgery: 26 (3.1%) during hospital stay, 61 (7.2%) between discharge and 3 months, and 34 (4.0%) between 3 and 6 months. Among patients with known fall status, 15 (6.9%) fallers were deceased, while no deaths occurred in the non-faller group. In the group with unknown fall status, 107 patients (27.9%) were deceased.

Age, BMI, gender, and ASA classification were similar between groups (Table 1). The pre-fracture mobility score showed that 27.4% of fallers walked without aids, compared to 59.2% of non-fallers and 27.1% of the unknown group. The activities of daily living, measured with the Katz ADL, showed that 47.5% of fallers were (nearly) independent with a score of 0 or 1, while 76.3% of non-fallers were (nearly) independent. In the unknown group, 48.7% were (nearly) independent. Before hemiarthroplasty, 53.4% of fallers lived independently at home, compared to 80.8% of non-fallers and 48.7% of the unknown group. Additionally, 26.0% of fallers were diagnosed with suspected or evident dementia, compared to 6.7% of non-fallers and 29.4% of the unknown group.

**Table 1:** Baseline characteristics of the study population.

	Fallers n = 219	Non-fallers n = 240	Unknown n = 384
**Mean (SD)**			
Age, years	82.4 (7.9)	80.2 (7.1)	83.2 (7.5)
BMI, kg/m^2^	24.2 (4.0)	25.0 (3.8)	24.4 (4.1)
Length of acute hospital stay, days	8.4 (5.8)	7.0 (3.5)	8.2 (6.6)
**N (%)**			
*Gender*			
Female	147 (67.1%)	155 (64.6%)	243 (63.3%)
Male	72 (32.9%)	85 (35.4%)	141 (36.7%)
*Polypharmacy*			
≥ 5 medications	148 (67.6%)	136 (56.7%)	274 (71.4%)
*Mobility before femoral neck fracture*			
Mobile without aids	60 (27.4%)	142 (59.2%)	104 (27.1%)
Mobile outdoors with 1 cane	32 (14.6%)	18 (7.5%)	39 (10.2%)
Mobile outdoors with 2 canes or walker	91 (41.6%)	49 (20.4%)	151 (39.3%)
Somewhat mobile indoor	22 (10.0%)	12 (5.0%)	41 (10.7%)
No functionality of lower extremity	2 (0.9%)	2 (0.8%)	6 (1.6%)
*Katz ADL score*			
0 (independent)	75 (34.2%)	148 (61.7%)	130 (33.9%)
1	29 (13.2%)	35 (14.6%)	57 (14.8%)
2	26 (11.9%)	8 (3.3%)	32 (8.3%)
3	18 (8.2%)	4 (1.7%)	40 (10.4%)
4	13 (5.9%)	5 (2.1%)	17 (4.4%)
5	11 (5.0%)	3 (1.3%)	10 (2.6%)
6 (dependent)	4 (1.8%)	0 (0%)	17 (4.4%)
*Living situation*			
Home and independent	117 (53.4%)	194 (80.8%)	186 (48.4%)
Home with daily/ADL assistance	60 (27.4%)	24 (10%)	97 (25.7%)
Assisted living facility	25 (11.4%)	11 (4.6%)	41 (10.9%)
Rehabilitation center	2 (0.9%)	0 (0%)	7 (1.8%)
Nursing home	13 (5.9%)	10 (4.2%)	46 (12.2%)
*ASA score*			
1	7 (3.2%)	8 (3.3%)	8 (2.1%)
2	80 (36.5%)	99 (41.3%)	107 (27.9%)
3	111 (50.7%)	116 (48.3%)	226 (58.9%)
4	13 (5.9%)	7 (2.9%)	23 (6.0%)
*Surgical approach*			
Direct Lateral Approach (DLA)	117 (53.4%)	127 (52.9%)	213 (55.5%)
Posterolateral Approach (PLA)	102 (46.6%)	113 (47.1%)	171 (44.5%)
Complications during hospital stay	82 (37.4%)	60 (25%)	158 (41.1%)
*Discharge destination*			
Home and independent	9 (4.1%)	16 (6.7%)	9 (2.3%)
Home with daily/ADL assistance	41 (18.7%)	87 (36.3%)	59 (15.4%)
Assisted living facility	16 (7.3%)	6 (2.5%)	30 (7.8%)
Rehabilitation center	119 (54.3%)	116 (48.3%)	192 (50.0%)
Nursing home	32 (14.6%)	14 (5.8%)	60 (15.6%)
Hospice	0 (0%)	0 (0%)	2 (0.5%)
*Cardiovascular comorbidity*			
Ischemia, heart failure, arrhythmia	100 (45.7%)	107 (44.6%)	200 (52.1%)
*Neurological comorbidity*			
CVA, TIA, Parkinson, epilepsy	75 (34.2%)	52 (21.7%)	111 (28.9%)
*Pulmonary comorbidity*			
COPD, asthma, pulmonary embolism	44 (20.1%)	33 (13.8%)	80 (20.8%)
Dementia (suspected or evident)	57 (26.0%)	16 (6.7%)	113 (29.4%)

BMI, Body Mass Index; ADL, Activities in Daily Living; ASA, American Society of Anaesthesiologists Physical Status (1 = Healthy, 2 = Mild disease, 3 = Severe disease, 4 = Life-threatening); CVA, Cerebral Vascular Accident; TIA, Transient Ischemic Attack; COPD, Chronic Obstructive Pulmonary Disease.

### Time to first fall, number of falls and injuries

**Fig. 2:**
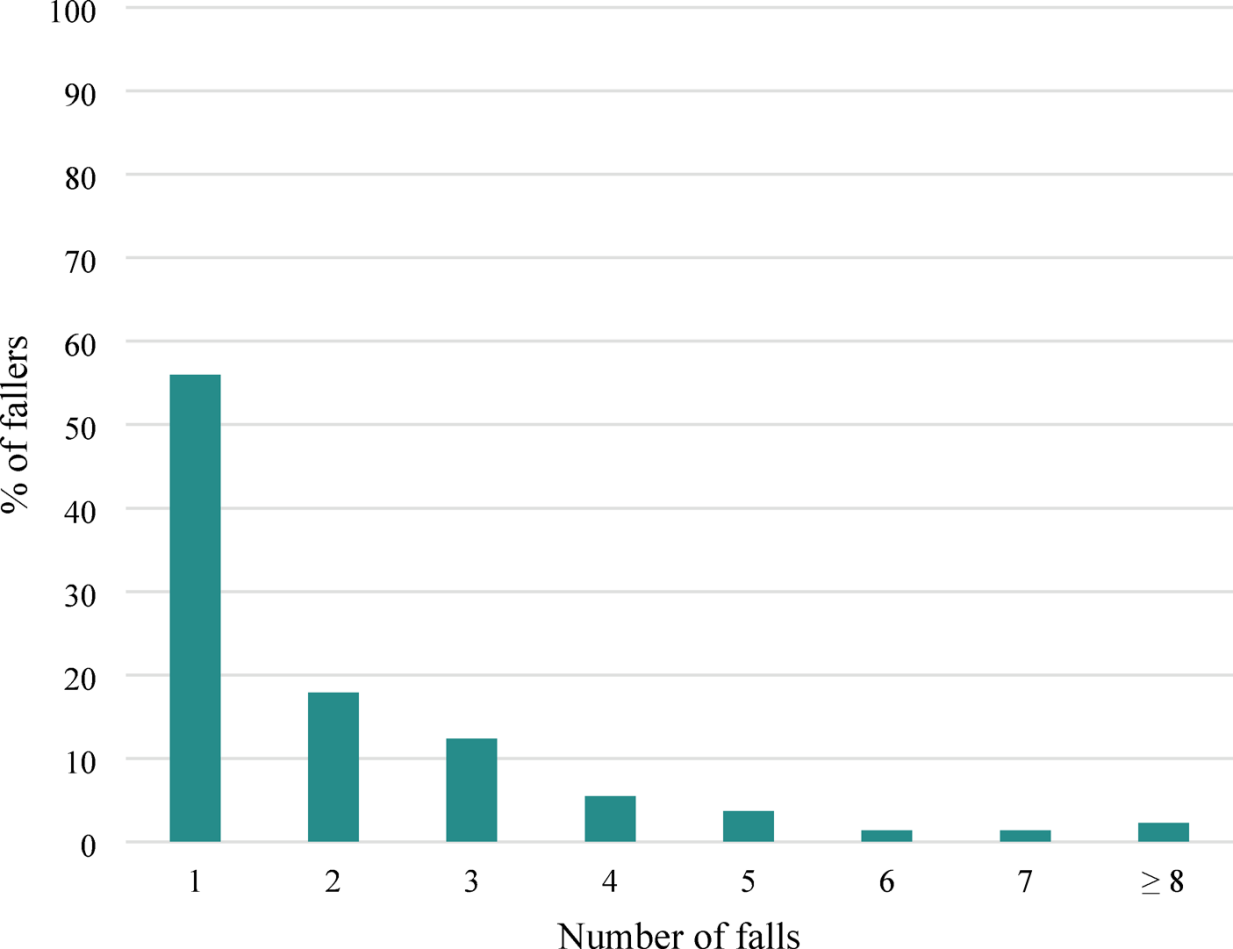
Number of falls within 6 months after hemiarthroplasty. This bar chart shows the percentage of fallers based on the number of falls experienced within 6 months after hemiarthroplasty. The x-axis represents the number of falls (1, 2, 3, 4, 5, 6, 7, ≥ 8), and the y-axis represents the percentage of fallers, ranging from 0 to 100%. The majority of fallers experienced only 1 fall, with a decline in the percentage of fallers as the number of falls increases.

Among the 219 fallers, 21 patients (9.6%) fell during their hospital admission, while 107 patients (48.9%) fell for the first time between discharge and 3 months after hemiarthroplasty surgery. Additionally, 91 patients (41.6%) experienced their first fall between 3 and 6 months post-surgery. Nearly 20% of the fallers (39 patients) fell twice during the study period, and 12.3% (27 patients) fell three times within 6 months after surgery (Fig. 2). In total, these 219 patients reported 474 falls within 6 months after hip hemiarthroplasty. Additionally, 34% of the fallers (75 patients) reported injuries as a result of their fall, such as fractures, dislocations or brain contusions.

### Predictors for falling

The univariable analysis (Table 2) showed that falling was significantly associated with seven predictors collected before hemiarthroplasty surgery: higher age, polypharmacy (e.g. ≥ 5 medications), impaired mobility, higher Katz ADL score indicating more difficulties in daily activities, not living independently, having neurological comorbidity and dementia. Falling was also significantly associated with three predictors collected at discharge: longer hospital stay, in-hospital complications and being discharged to a more dependent living situation.

**Table 2:** Univariable analysis of predictors for falling after hemiarthroplasty.

	**Univariable model**		
**Pre-surgery predictors**	**Odds ratio**	**95% CI**	**P value**
Age, years	1.04	1.02 – 1.07	0.002
BMI, kg/m^2^	0.95	0.91 – 1.01	0.076
Polypharmacy, ≥ 5 medications	1.61	1.09 – 2.37	0.016
Gender, male	1.12	0.76 – 1.65	0.567
Mobility	1.82	1.51 – 2.20	< 0.001
Katz ADL score, score 0–6	1.74	1.46 – 2.08	< 0.001
Living situation, dependent	1.78	1.40 – 2.26	< 0.001
ASA score	1.24	0.92 – 1.66	0.158
Cardiovascular comorbidity	1.05	0.72 – 1.51	0.817
Neurological comorbidity	1.88	1.24 – 2.85	0.003
Pulmonary comorbidity	1.58	0.99 – 2.59	0.071
Dementia	5.02	2.78 – 9.06	< 0.001
**Pre-discharge predictors**			
Length of acute hospital stay, days	1.08	1.03 – 1.13	0.002
Surgical approach, PLA	0.98	0.68 – 1.41	0.913
In-hospital complications	1.80	1.21 – 2.69	0.004
Discharge destination	1.24	1.09 – 1.41	0.001

Significance level for univariable analysis: p < 0.05. Abbreviations: BMI, Body Mass Index; ADL, Activities in Daily Living; ASA, American Society of Anaesthesiologists Physical Status; PLA, Posterolateral Approach.

#### Predictors collected before hemiarthroplasty surgery

No significant multicollinearity (r > 0.6 and VIF ≤ 2.1) was detected among the predictors; therefore, all candidate predictors were used in the multivariable logistic regression analysis. Using backward selection with a p-value threshold of 0.157, the analysis identified five relevant pre-surgery predictors of falling: impaired mobility, ADL difficulties, neurological comorbidity, pulmonary comorbidity, and dementia. These results are summarized in Table 3. The odds ratio for mobility was 1.25, indicating that for each point increase in the mobility score (worse mobility), the likelihood of falling increased by 24.7%. The odds ratio for a higher Katz ADL score was 1.51, suggesting that each unit increase in the Katz ADL score (i.e. more difficulties in the activities of daily living) raised the odds of falling by 51.4%. Patients with neurological comorbidity had an odds ratio of 1.80, implying a 80.1% increased risk of falling. For pulmonary comorbidity, the odds ratio was 1.90, indicating an 90.2% higher likelihood of falling. Lastly, the odds ratio for dementia was 2.02, showing that dementia doubles the odds (101.6% higher) of falling compared to those without dementia. The multivariable model, utilizing predictors collected pre-surgery, exhibited an R^2^ of 0.22 and an AUC of 0.73 (95% CI 0.68–0.78).

**Table 3:** Multivariable analyses of predictors for falling after hemiarthroplasty.

	**Multivariable model pre-surgery**		
**Pre-surgery predictors**	**Odds ratio**	**95% CI**	**P value**
Mobility	1.25	0.96 – 1.62	0.095
Katz ADL score	1.51	1.16 – 1.98	0.002
Neurological comorbidity	1.80	1.02 – 3.18	0.043
Pulmonary comorbidity	1.90	0.97 – 3.72	0.061
Dementia	2.02	0.80 – 5.11	0.140
**Pre-discharge predictors**	**Multivariable model pre-discharge**		
Mobility	1.24	0.94 – 1.63	0.135
Katz ADL score	1.74	1.33 – 2.26	< 0.001
In-hospital complications	2.10	1.16 – 3.82	0.015

Significance level for multivariable analyses: p < 0.157. ADL, Activities in Daily Living. Pre-surgery model: R^2^ = 0.217, AUC = 0.730; discharge model: R^2^ = 0.216, AUC = 0.709.

#### Predictors collected at discharge

Of all the variables collected at discharge, the multivariable logistic regression analysis showed that falling was significantly associated with three variables: impaired mobility (OR 1.24, 95%CI 0.94–1.63), indicating that each point increase in the mobility score (worse mobility) increased the odds of falling by 23.6%; ADL difficulties (OR 1.74, 95%CI 1.33–2.26), suggesting that each unit increase in the Katz ADL score (more difficulties in daily activities) raised the odds of falling by 73.7%; and the presence of in-hospital complications (OR 2.10, 95%CI 1.16–3.82), indicating that experiencing in-hospital complications more than doubled the odds of falling (110.4% higher). This model, incorporating predictors collected at discharge, demonstrated an R^2^ of 0.22 and an AUC of 0.71 (95% CI 0.66–0.76). These results indicate that adding variables collected at discharge did not significantly enhance the model’s predictive power, both models displayed moderate discriminative ability in identifying patients at risk of falling within 6 months after hip hemiarthroplasty.

## Discussion

In this study, out of 459 patients with known fall status, 219 (48%) experienced at least one fall during the 6-month follow-up. Furthermore, it is noteworthy that patients in the ‘unknown’ group closely resemble the ‘fallers’ group regarding baseline characteristics. The high mortality (27.9%) in the unknown group may reflect a competing risk, as patients who died could no longer experience falls. Thus, the number of falls in this study might underestimate the magnitude of the problem of fall incidents and highlights the importance of initiating fall prevention measures immediately after a hip fracture to potentially prevent subsequent falls. The world fall guidelines recommend offering a multicomponent fall analysis to every older adult experiencing a fall with such injury^21^. Subsequent falls have great consequences for patients, not only the injury itself, but also the loss of independence as the result of the fall, directly due to the injury, or indirect due to fear of falling. Although 34% of the fallers reported serious injuries, fall prevention is also crucial for those without injuries. Fall prevention should therefore be the second goal for every patient and healthcare professional after fixing the fracture.

Falling was associated with five pre-surgery variables: impaired mobility, ADL difficulties, neurological comorbidities, pulmonary comorbidities, and dementia. Our finding that impaired mobility is a predictor for falling is in line with the studies of Stewart et al.^12^ and Larrainzar-Garijo et al.^13^. Literature suggests that sarcopenia and muscle weakness are the underlying causes for this in older adults^22,23^, particularly evident in patients recovering from hemiarthroplasty. Furthermore, we found an association between ADL difficulties and an increased fall risk, which is similar to the observations of van Helden et al.^24,25^. We hypothesized that neurological comorbidity increases fall risk due to impaired balance and coordination. Pulmonary comorbidity may also contribute, as shortness of breath could lead to instability, fatigue, or reduced physical capacity, increasing the likelihood of falls. Suspected or evident dementia as a predictor for falling is partly in line with the study of Stewart et al.^12^, in which patients who fell exhibited lower cognitive scores. Furthermore, dementia might be an essential predictor of falling because it often exacerbates cognitive impairment and disorientation. Although dementia was identified as a significant predictor in the univariate analysis, it did not remain in the pre-discharge model, likely due to its partial association with other variables such as mobility and ADL difficulties. Multicollinearity was checked in two ways, but no correction was necessary. Of the variables collected at discharge, falling was additionally significantly associated with in-hospital complications. This is a novel finding, emphasizing the importance of preventing in-hospital complications, as they represent a modifiable risk factor for falling. When complications do occur, it is important to recognize that patients are likely to be discharged in a weakened state and may require better post-discharge support and guidance to prevent further falls.

The use of both pre-operative and post-operative variables allowed us to develop prediction models for different stages of patient management. The pre-operative model can guide surgical decision-making and help select appropriate interventions before surgery, while the post-operative model can inform decisions about whether to discharge a patient home or to a rehabilitation center with additional support. We used the Akaike Information Criterion (AIC) to guide predictor selection. By penalizing overly complex models, AIC reduces the risk of overfitting, ensuring that the selected predictors are truly informative. This approach allows to retain variables that are not only statistically relevant but can also serve as individual indicators or “red flags” at critical points in the patient’s healthcare journey.

A strength of our study is the large, multicenter cohort, providing a representative sample of the hip fracture population. This is particularly evident in the participation of patients with dementia, a group often excluded from studies. Additionally, the use of data from a prospective study helps avoid the considerable underreporting of falls seen in retrospective cohorts.

However, there are some limitations to our study. First, we do not know whether patients from the unknown group experienced additional falls after hemiarthroplasty, since the APOLLO trial was not originally designed to assess fall risk. Although these patients were excluded from the regression analyses, they were intentionally retained in the baseline characteristics table to allow comparison with fallers and non-fallers. This group closely resembled the fallers in terms of baseline characteristics and had a higher mortality rate, suggesting that the true incidence of falls may be underestimated due to competing risk by mortality. Second, the use of surveys in this population may introduce recall bias, especially given the length of time between surgery and the survey, which could affect patients’ ability to accurately recall falls. We mitigated this by checking all medical health records for admissions due to a fall. Despite this, it is possible that patients were admitted to other hospitals, leading to underreporting of the actual number of falls. Third, some clinically relevant confounders were not available in the APOLLO dataset, including prior fall history and detailed information on high-risk medications (e.g., sedatives, hypnotics, antidepressants). Their omission may have resulted in residual confounding, potentially inflating or attenuating the observed associations between impaired mobility, ADL difficulties, comorbidities and fall risk; thus, findings should be interpreted as associations rather than causal effects.

In conclusion, the high incidence of falls within 6 months after hip hemiarthroplasty for acute femoral neck fracture is a significant concern. This finding underscores the necessity for all physicians involved in treating hip fractures to recognize the importance of fall prevention afterwards. We identified predictors for falling, including pre-surgery factors such as impaired mobility, ADL difficulties, and dementia. Although fall prevention strategies have demonstrated success in other populations, their effectiveness for this specific group remains to be validated. Therefore, further research should focus on validating prediction models and evaluating the effectiveness of fall prevention strategies. A hybrid implementation-effectiveness trial should be conducted to determine if and which fall prevention strategy can be effectively implemented and lead to a reduction in falls in patients with a hip fracture.

## Supplementary Information

Below is the link to the electronic supplementary material.

Supplementary Material 1

## Data Availability

The metadata of the APOLLO trial dataset are publicly available through the FAIR (Findable, Accessible, Interoperable, and Reusable) data repository at 10.34894/K99WGS. Due to privacy and ethical restrictions, the underlying data cannot be made fully open but is available from the corresponding author upon reasonable request, in accordance with institutional and ethical regulations. The repository provides the persistent identifier, metadata description, and access procedure required for requesting the dataset.

## References

[1] The World Health Organization (WHO). Falls: key facts. *WHO* http://www.who.int/news-room/fact-sheets/detail/falls (September 2024, last accessed).

[2] The World Health Organization (WHO). *WHO Global Report on Falls Prevention in Older Age. WHO* https://extranet.who.int/agefriendlyworld/wp-content/uploads/2014/06/WHo-Global-report-on-falls-prevention-in-older-age.pdf (3 September 2024, last accessed).

[3] Souza, A. Q. et al. Incidence and predictive factors of falls in community-dwelling elderly: A longitudinal study. *Cien. Saude Colet.* **24**, 3507–3516 (2019). 31508768 10.1590/1413-81232018249.30512017

[4] Chen, W. C. et al. The relationship between falling and fear of falling among community-dwelling elderly. *Medicine (Baltimore)* **100**, e26492. 10.1097/MD.0000000000026492 (2021). 34190176 10.1097/MD.0000000000026492 PMC8257838

[5] Gazibara, T. et al. Falls, risk factors and fear of falling among persons older than 65 years of age. *Psychogeriatrics* **17**, 215–223 (2017). 28130862 10.1111/psyg.12217

[6] Galet, C. et al. Fall injuries, associated deaths, and 30-day readmission for subsequent falls are increasing in the elderly US population: a query of the WHO mortality database and National Readmission Database from 2010 to 2014. *Clin. Epidemiol.* **10**, 1627–1637 (2018). 30519111 10.2147/CLEP.S181138 PMC6233862

[7] Schene, M. R. et al. The “Can Do, Do Do” framework applied to assess the association between physical capacity, physical activity and prospective falls, subsequent fractures, and mortality in patients visiting the fracture liaison service. *J. Pers. Med.* **14**, 337 (2024). 38672964 10.3390/jpm14040337 PMC11050804

[8] Schene, M. R., Wyers, C. E., Driessen, A. M. H. *et al.* Imminent fall risk after fracture. *Age Ageing* **52**, afad201 (2023). 10.1093/ageing/afad201 10.1093/ageing/afad201 37930741

[9] Miller, R. R. et al. Repeat falls and the recovery of social participation in the year post-hip fracture. *Age Ageing* **38**, 570–575 (2009). 19586976 10.1093/ageing/afp107 PMC2981467

[10] Pils, K. et al. Predictors of falls in elderly people during rehabilitation after hip fracture—who is at risk of a second one?. *Z. Gerontol. Geriatr.* **36**, 16–22 (2003). 12616403 10.1007/s00391-003-0142-9

[11] Schroder, H. M., Petersen, K. K. & Erlandsen, M. Occurrence and incidence of the second hip fracture. *Clin. Orthop. Relat. Res.* **289**, 166–169 (1993). 8472408

[12] Stewart, A. et al. Risk factors associated with increased falls in a hip fracture population. *Gerontology* **45**, 233 (1999). 10394082 10.1159/000022093

[13] Larrainzar-Garijo, R. et al. Risk factors for a second nonsimultaneous hip fracture in a prospective cohort study. *Arch. Orthop. Trauma Surg.* **142**, 2611–2617 (2022). 34125250 10.1007/s00402-021-03991-0

[14] Vranken, L. et al. Association between incident falls and subsequent fractures in patients attending the fracture liaison service after an index fracture: a 3-year prospective observational cohort study. *BMJ Open* **12**, e058983. 10.1136/bmjopen-2021-058983 (2022). 35896286 10.1136/bmjopen-2021-058983 PMC9335024

[15] Tol, M. et al. Posterolateral or direct lateral surgical approach for hemiarthroplasty after a hip fracture: a randomized clinical trial alongside a natural experiment. *JAMA Netw. Open* **7**, e2350765. 10.1001/jamanetworkopen.2023.50765 (2024). 38206628 10.1001/jamanetworkopen.2023.50765 PMC10784859

[16] Katz, S. & Akpom, C. A. A measure of primary sociobiological functions. *Int. J. Health Serv.* **6**, 493–508 (1976). 133997 10.2190/UURL-2RYU-WRYD-EY3K

[17] Peduzzi, P. et al. A simulation study of the number of events per variable in logistic regression analysis. *J. Clin. Epidemiol.* **49**, 1373–1379 (1996). 8970487 10.1016/s0895-4356(96)00236-3

[18] Moons, K. G. et al. Transparent reporting of a multivariable prediction model for individual prognosis or diagnosis (TRIPOD): explanation and elaboration. *Ann. Intern. Med.* **162**, W1-73. 10.7326/m14-0698 (2015). 25560730 10.7326/M14-0698

[19] Sutherland, C. et al. Practical advice on variable selection and reporting using Akaike information criterion. *Proc. Biol. Sci.* **290**, 20231261. 10.1098/rspb.2023.1261 (2023). 37752836 10.1098/rspb.2023.1261 PMC10523071

[20] Heinze, G., Wallisch, C. & Dunkler, D. Variable selection—A review and recommendations for the practicing statistician. *Biom. J.* **60**, 431–449 (2018). 29292533 10.1002/bimj.201700067 PMC5969114

[21] Kwan, E. & Straus, S. E. Assessment and management of falls in older people. *CMAJ* **186**, E610–E621. 10.1503/cmaj.131213 (2014). 24982291 10.1503/cmaj.131330 PMC4216276

[22] Yeung, S. S. Y. et al. Sarcopenia and its association with falls and fractures in older adults: a systematic review and meta-analysis. *J. Cachexia Sarcopenia Muscle* **10**, 485–500 (2019). 30993881 10.1002/jcsm.12411 PMC6596401

[23] Moreland, J. D. et al. Muscle weakness and falls in older adults: a systematic review and meta-analysis. *J. Am. Geriatr. Soc.* **52**, 1121–1129 (2004). 15209650 10.1111/j.1532-5415.2004.52310.x

[24] van Helden, S. et al. Risk of falling in patients with a recent fracture. *BMC Musculoskelet. Disord.* **8**, 55 (2007). 17598891 10.1186/1471-2474-8-55 PMC1933426

[25] van Helden, S. et al. Bone and fall-related fracture risks in women and men with a recent clinical fracture. *J. Bone Joint Surg. Am.* **90**, 241–248 (2008). 18245581 10.2106/JBJS.G.00150

